# A Diagnostic Challenge for Bilateral Lung Involvement in a 13‐Month‐Old Saudi Child Due to Multiple Popcorn Kernel Aspirations: A Case Report

**DOI:** 10.1002/ccr3.70276

**Published:** 2025-03-03

**Authors:** Mohamed Al Omari, Saleh Al Fulayyih, Ahmed Nawfal M. Alshammari, Sara Amer Alomar, Majid Alhumaidi S. Aldossary, Maan Abdullah Albehair, Mohammed Shahab Uddin

**Affiliations:** ^1^ Department of Pediatric Ministry of National Guard Health Affairs Dammam Saudi Arabia; ^2^ Department of Otorhinolaryngology Ministry of National Guard Health Affairs Dammam Saudi Arabia

**Keywords:** diagnostic errors, foreign bodies, pediatric emergency medicine, popcorn, respiratory aspiration, Saudi Arabia

## Abstract

Foreign body aspiration (FBA) should be considered in the differential diagnosis of pediatric respiratory cases, particularly when symptoms are atypical and bilateral lung involvement is present. Early recognition and prompt intervention are crucial to preventing severe complications.

## Graphical abstract

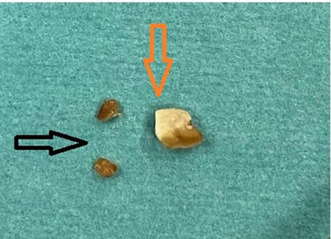

## 1 Background

Foreign body aspiration (FBA) is a leading cause of unintentional home mortality among children under 5 years of age, with fatality rates between 2.1% and 6.2% in the international literature [1, 2, 3]. Although preventable, FBA poses a significant financial burden, accounting for approximately 6.6% of pediatric admissions per 10,000 pediatric patient days [4, 5]. Common complications of undetected FBA include pneumonia, atelectasis, granulomas, broncho‐esophageal fistulas, and bronchiectasis, which can lead to permanent lung damage [6, 7, 8, 9, 10, 11, 12, 13]. The incidence of these complications ranges from 14.6% to 27.8% globally [11, 14, 15].

Diagnosing FBA can be challenging, especially when it is based on complex medical histories and nonspecific symptoms. Often, the absence of parents during a choking episode makes it difficult for physicians to identify the initial choking crisis. Additionally, the radiolucency of aspirated materials further complicates the diagnosis [16, 17, 18, 19, 20]. Common misdiagnoses include bronchial asthma, pneumonia, croup infection, and acute bronchiolitis [11, 21, 22, 23, 24, 25, 26].

It is not uncommon for children to experience asymptomatic periods following aspiration, which can delay diagnosis [27, 28]. Research indicates that approximately 82% of children in the late‐diagnosis group were diagnosed more than 7 days after symptom onset, compared to 25% in the early diagnosis group who were diagnosed within 7 days [20]. In a study by Mu et al., 95% of late‐diagnosed cases were identified more than a month after the initial event, and some cases took years to diagnose accurately [29, 30].

In Saudi Arabia, aspirated objects frequently include fish bones, alfalfa and Lucerne seeds, peanuts, and watermelon seeds [31, 32]. Notably, there are few reports of popcorn inhalation among Saudi children, with limited mention in the international literature [33, 34]. This study aimed to enhance Saudi parents’ awareness regarding the hazards of various foreign bodies, including popcorn, and to address knowledge gaps among general practitioners and pediatricians concerning the risks of popcorn aspiration in children. This knowledge could facilitate more rapid intervention. A noteworthy case involved a 13‐month‐old baby who was found to have multiple popcorn kernels lodged in both main bronchi, highlighting an exceptional case scenario.

## 2 Case History/Examination

A previously healthy 13‐month‐old Saudi boy presented to the emergency department with symptoms of fever and cough, persisting for 2 days. During episodes of coughing, his mother noticed his lips appeared darker, which raised concerns about a possible SARS‐CoV‐2 infection. However, there were no known exposures to the virus. The child had no history of asthma or chronic illnesses and no significant birth or family medical history. On examination, he appeared calm and comfortable, resting quietly in bed. His vital signs were as follows: a body temperature of 37.2°C, a heart rate of 110 beats per minute, a respiratory rate of 38 breaths per minute, and oxygen saturation at 100% on room air. Physical examination showed mild suprasternal and subcostal retractions. His breath sounds were reduced bilaterally, with scattered crepitations noted in the left lung.

## 3 Methods

### 3.1 Differential Diagnosis, Investigations, and Treatment

**FIGURE 1:**
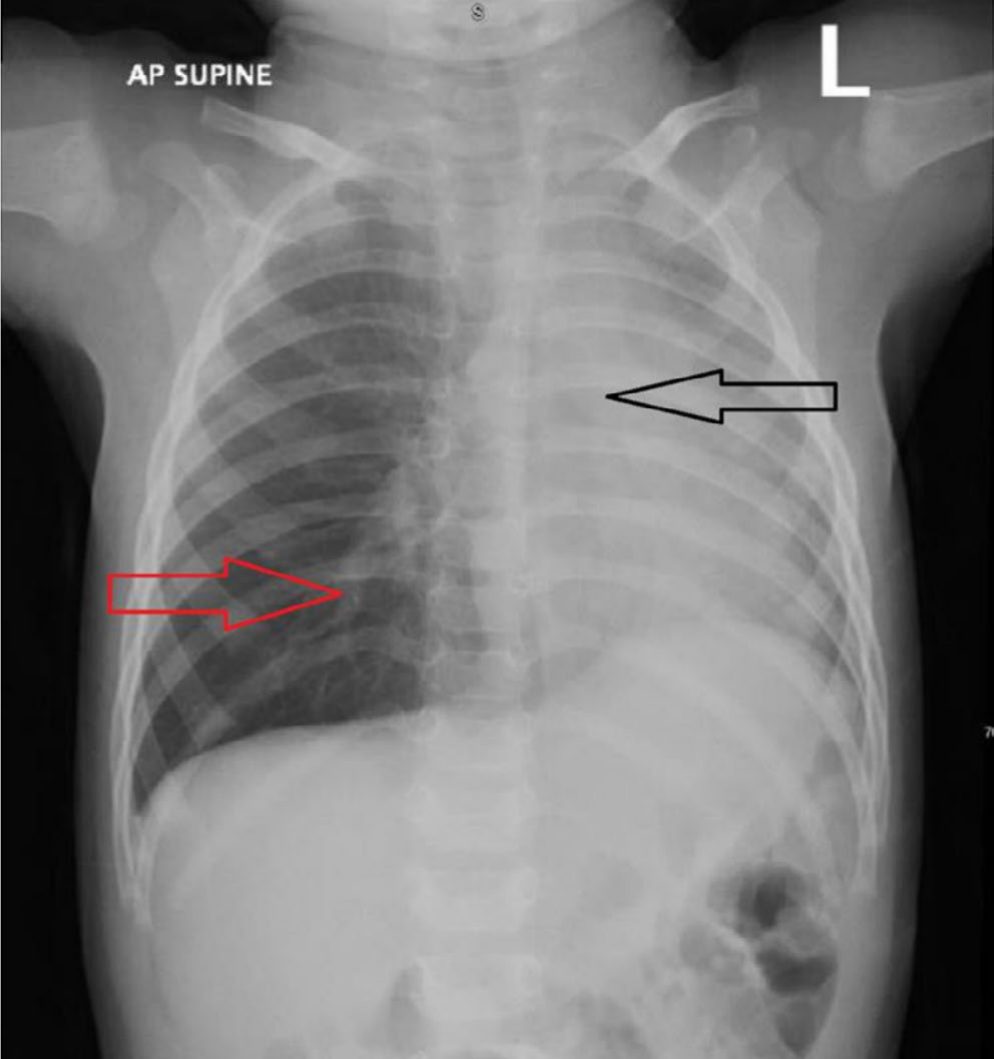
Anteroposterior (AP) supine chest radiograph of a pediatric patient on admission. The black arrow highlights hyperinflation in the right lower lobe of the lung, whereas the red arrow indicates opacity in the left lower lobe, suggestive of potential pathological changes such as pneumonia or atelectasis.

**FIGURE 2:**
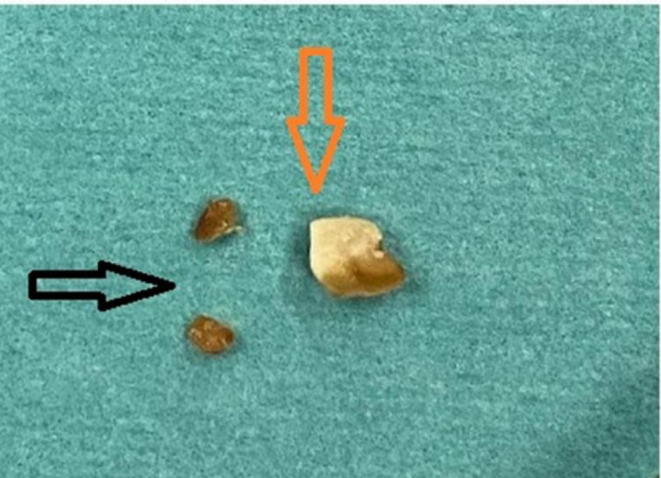
showing multiple popcorn kernels extracted post‐bronchoscopy. The orange arrow indicates a larger kernel with a notable color change due to prolonged retention, while the black arrow points to smaller kernels that exhibit more discoloration. One popcorn was removed from the Right (large), and two more from the left bronchus.

The initial differential diagnosis was viral pneumonia, bacterial pneumonia, bronchiolitis, and foreign body aspiration. The presence of a viral infection, together with respiratory symptoms, prompted the treatment of pneumonia. A chest X‐ray showed a left‐sided mediastinal shift, right lung hyperinflation, and left lung consolidation or collapse (Figure 1). Laboratory studies revealed normal electrolytes, inflammatory markers, and liver and kidney function tests. The RT‐PCR for SARS‐CoV‐2 was negative, but the adenovirus PCR respiratory panel was positive (Table 1). The patient's pneumonia was treated with salbutamol nebulization and intravenous cefuroxime (50 mg/kg every 8 h). The acute onset of respiratory symptoms in a previously healthy child included abnormal findings like minor retractions, asymmetrically reduced breath sounds, and weak crepitations on physical examination. Chest X‐ray revealed a left‐sided mediastinal shift, right lung hyperinflation, and left lung collapse, (Figure 1) indicating an obstructive pathology. Additionally, the absence of known SARS‐CoV‐2 exposure and a negative RT‐PCR test further shifted the suspicion away from infectious causes. Persistent asymmetry in lung function despite treatment raised further concern for a mechanical obstruction, culminating in the decision to perform bronchoscopy by an ENT specialist, which confirmed the presence of foreign bodies. Multiple popcorn kernels were retrieved from both the right and left bronchi, with some obstructing the main bronchi and acting as one‐way valves, causing air entrapment and hyperinflation in the right lung and collapse in the left (Figure 2).

**TABLE 1:** Baseline laboratory data of the patient upon admission.

	Result	Reference
Hb	11.3 g/L	10.6–14.5 g/L
WBC	9.0 × 10^9^/L	6–16 × 10^9^/L
Platelets	444 × 10^9^/L	150–450 × 10^9^/L
CRP	2.7 g/L	5 g/L
Sodium	136 μmol/L	138–145/ μmol L
Potassium	4.6 μmol/L	3.4–4.7 μmol/L
Chloride	105 μmol/L	95–110 μmol/L
Bun	3.6 μmol/L	1.8–6 μmol/L
Creatinine	38 μmol/L	27–62 μmol/L
Calcium	2.5 μmol/L	2.25–2.75 μmol/L
RT‐PCR Covid‐19	Negative	x
PCR Respiratory panel multiplex	Adenovirus	x

*Note:* The values are compared to reference ranges.

Abbreviations: CRP, C‐reactive protein; Hb, hemoglobin; RT‐PCR, reverse transcriptase polymerase chain reaction; WBC, white blood cells.

### 3.2 Conclusions and Results (Follow‐Up)

**FIGURE 3:**
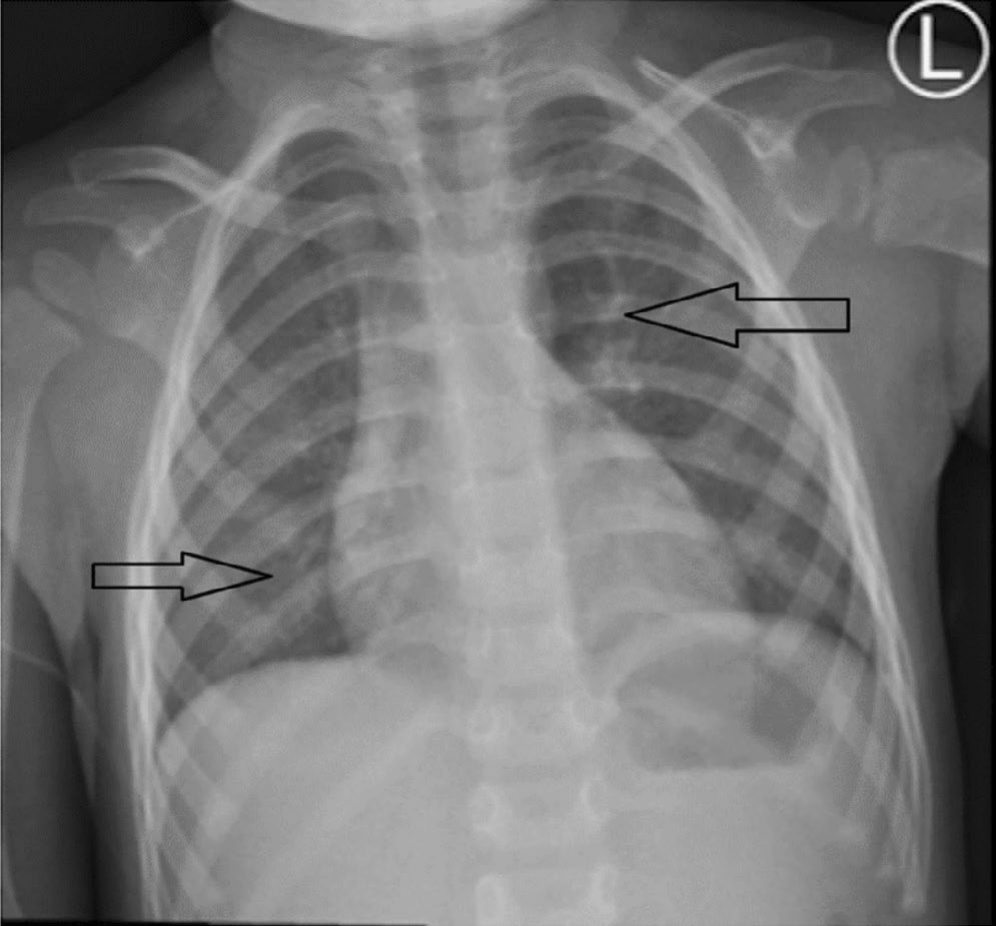
X‐ray chest PA views post bronchoscopy, demonstrating Right lung field Hyperinflation (arrow) resolved secondary to removal of popcorn which is partial obstruction of the right main bronchus. Left lung collapse resolved (arrow) after removal of two popcorns from the Left main bronchus.

Following the removal of the foreign bodies, the patient's airway was cleaned, resulting in the remission of symptoms and the restoration of lung function. A subsequent chest X‐ray verified the resolution of right lung hyperinflation and left lung collapse (Figure 3). The patient remained stable throughout and after the treatment, and she was discharged in good health with orders to return in 4 weeks. This case highlights the diagnostic challenges of bilateral lung involvement caused by popcorn kernel aspiration in young children, emphasizing the importance of thorough clinical evaluation, chest X‐ray interpretation, and awareness of the unique risks of popcorn aspiration to avoid misdiagnosis and complications.

## 4 Discussion

Foreign body aspiration (FBA) is common and often poses diagnostic challenges for pediatricians [35]. In many cases, including ours, FBA is misdiagnosed as pneumonia or acute bronchiolitis [8, 19, 36]. Our patient had been coughing up popcorn crumbs for 2 days prior to presentation but was otherwise healthy until he contracted the flu. The initial denial of sudden onset of coughing by the parents, combined with the added viral infection, diverted the doctor's attention. Our experience aligns with findings from Ulas et al. (2022), which indicate that cough (83.9%) and wheezing (59.9%) are prevalent symptoms of FBA, emphasizing the need for heightened clinical suspicion [37]. Moreover, studies indicate that the primary cause of FBA is parental ignorance of the risks associated with feeding small organic foods to young infants [36, 38]. During medical history collection, crucial details of choking and coughing after eating were either ignored or overlooked [9]. Notably, FBA sometimes presents with no symptoms [39]. Patients may feel fine shortly after aspiration, delaying diagnosis.

Large‐scale cohort studies on FBA diagnostic errors have highlighted the prevalence of misdiagnosis. Of the 226 cases, 80 (35.4%) were initially misdiagnosed. Among these, 51.3% of the children initially diagnosed with bronchiolitis later received a different diagnosis. Most of these diagnostic errors (76.3%) were due to mistakes in test ordering, execution, and interpretation, with difficulties or delays in obtaining crucial case history information being the second most common cause (20%) [36, 40, 41].

In our case, the doctors initially misinterpreted the chest radiograph, and there was a failure or delay in obtaining a critical case history. Previous studies have indicated that an adequate diagnosis of FBA could take days, months, or even years [6, 20, 29, 30, 31, 32]. Ulas et al. point out that radiological findings in children are frequently less apparent than in adults, with a significant portion of chest X‐rays appearing normal even when foreign bodies are present [37].

Remarkably, our patient's diagnosis was made within 6 h of admission, which is a notable achievement. Some studies define “early diagnosis” as performing bronchoscopy within 24 h of suspecting aspiration or noticing the first signs of possible aspiration [27]. As indicated by Ulas et al., early bronchoscopy, ideally within 24 h of symptom onset, is crucial and can prevent severe complications such as prolonged hospitalization and chronic lung damage [37]. In our case, the popcorn kernels represented such a challenge, contributing to the bilateral lung involvement, which is rarely addressed in the literature but noted in the study by Ulas et al. The initial oversight could have led to severe outcomes if not for the timely bronchoscopic intervention [37].

Despite the initial delay in diagnosis, the child did not experience further complications, such as prolonged hospitalization, recurrent infections, bronchiectasis, long‐term lung damage, or death, which are often reported in the literature. Delayed diagnoses in one‐third of cases are attributed to parents disregarding medical advice or doctors misdiagnosing patients based on unclear clinical signs [8]. In this instance, the prolonged diagnosis period may have been due to parents’ lack of awareness regarding the seriousness of symptoms such as coughing and choking [35]. This supports our observation where the initial chest radiograph misinterpretation led to a delayed consideration of FBA, underscoring the necessity for improved radiographic assessment in suspected pediatric FBA cases [37].

Misinterpretation of chest X‐rays by emergency room physicians or pediatric residents‐in‐training is not uncommon. The absence of radiological reports during weekends exacerbates this issue. Given the significant financial and medical implications of adverse events, error reduction is a critical priority in healthcare [42]. Studies indicate that when emergency physicians, rather than board‐certified radiologists, reviewed chest radiographs, most errors occurred, with a reported error rate of 41.7% [43]. Conversely, the implementation of a quality emergency residency program has been shown to decrease errors by up to 4.8% [44]. In certain cases, there was as much as a 56% discrepancy between radiologists and emergency room doctors in the interpretation of chest radiographs [45]. Despite these challenges, radiologists in emergency settings must prioritize the reading of X‐rays from young children because of their potential impact on treatment decisions [43]. Continuous 24‐h availability of Emergency Room (ER) radiology services can significantly alter therapeutic approaches [46]. We advocate for enhanced radiologist availability to interpret ER chest films and for additional training of ER physicians in pediatric chest X‐ray interpretation. Such measures are essential for reducing diagnostic errors in Foreign Body Aspiration (FBA). Mastering chest X‐ray interpretation for FBA is vital for general pediatricians, especially when distinguishing these cases from those related to viral infections, which are often present concurrently. Enhancing the diagnostic accuracy of FBA is crucial under these circumstances.

Children around the globe frequently ingest foreign objects, a phenomenon that is often influenced by cultural differences in dietary habits. In the United States, foods commonly associated with foreign body aspiration (FBA) in children include peanuts, hard candies, meat, and bones [47, 48]. The European Survey on Foreign Body Injuries (ESFBI) reported that nuts, seeds, berries, corn, and beans are the most frequent culprits of such injuries [49]. In China, nuts and peanuts are regularly aspirated [20, 50, 51], whereas in India and South Africa, peanuts are the predominant foreign objects [52]. In Turkey, incidents involving nuts and seeds are common [53, 54]. This case is noteworthy because it documents the aspiration of “popcorn,” which deviates from the typically reported organic materials, thereby enriching our understanding of FBA in children. Uniquely, this case involved both lungs and had an atypical presentation of FBA. Although the signs of FBA are generally limited, bilateral lung involvement can occur because popcorn fragments can disperse and enter various parts of the airway. Our case study revealed that the mother was either unaware of or denied the presence of FBA symptoms. Such scenarios have been frequently discussed in the literature. For example, a survey involving 1490 mothers revealed that only 4.3% were aware that small toys could cause aspiration [55]. Moreover, 22% of the parents understood that consuming nuts, including peanuts, could lead to FBA in children. Additionally, 27.7% of the mothers were uncertain whether sudden coughing or choking episodes were indicative of FBA [56]. A recent survey found that many caregivers of young children also lacked awareness of FBA risks [57]. Pediatricians must exercise increased vigilance when diagnosing FBA in children. Indeed, enhancing parental awareness of FBA could lead to better preventative measures and outcomes.

## 5 Conclusion

In conclusion, our case reinforces the critical nature of considering FBA in differential diagnoses for pediatric patients presenting with respiratory symptoms and atypical signs. It highlights the need for public and professional education on the dangers of small, easily aspirated food items such as popcorn. Early recognition, accurate diagnosis, and prompt management are paramount to improving outcomes in pediatric FBA.

## Author Contributions

**Mohamed Al Omari:** conceptualization, investigation, writing – original draft. **Saleh Al Fulayyih:** methodology, writing – original draft, writing – review and editing. **Ahmed Nawfal M. Alshammari:** writing – original draft, writing – review and editing. **Sara Amer Alomar:** writing – review and editing. **Majid Alhumaidi S. Aldossary:** investigation, methodology, writing – original draft. **Maan Abdullah Albehair:** investigation, methodology, writing – original draft. **Mohammed Shahab Uddin:** conceptualization, data curation, formal analysis, methodology, supervision, visualization, writing – review and editing.

## Ethics Statement

The Ethics Committee waived ethical approval because the study was retrospective, and all therapies were provided as part of usual care. The submitted manuscript does not include any patient‐identifiable information, such as names, initials, hospital identification numbers, or photographs.

## Consent

The parents gave written informed consent to publish this report in accordance with the journal's patient consent policy.

## Conflicts of Interest

The authors declare no conflicts of interest.

## Data Availability

The data supporting the findings of this study are available upon request from the corresponding author. Due to privacy or ethical restrictions, the data are not publicly accessible.
